# Flexural Strength, Fatigue Behavior, and Microhardness of Three-Dimensional (3D)-Printed Resin Material for Indirect Restorations: A Systematic Review

**DOI:** 10.3390/ma18030556

**Published:** 2025-01-26

**Authors:** Cristian Abad-Coronel, Daniela Durán Urdiales, María Verónica Benalcázar Arias, Andrea Karina Córdova, María Sol Medina, Wilson Bravo Torres

**Affiliations:** Faculty of Dentistry, Universidad de Cuenca, Cuenca 010107, Ecuador; daniela.duran@ucuenca.edu.ec (D.D.U.); veronica.benalcazara@ucuenca.edu.ec (M.V.B.A.); andreak.cordova@ucuenca.edu.ec (A.K.C.); mariasol.medinaabad@edu.unife.it (M.S.M.); wilson.bravo@ucuenca.edu.ec (W.B.T.)

**Keywords:** 3D-printed composite resins, additive manufacturing, fatigue, flexural strength, mechanical properties

## Abstract

The purpose of this systematic review was to evaluate three mechanical properties of 3D-printed resins for indirect restorations according to published scientific evidence. This systematic review was conducted according to the PRISMA statement (preferred reporting elements for systematic reviews and meta-analyses). The search was performed by two investigators, (DD) and (VB), and a third (AC) resolved disagreements. Articles were searched in four digital databases: PubMed, EBSCO, Lilacs, and Science Direct, starting on 18 February 2024. As 3D-printing technology has shown significant advances in the last 5 years, the review was conducted with a publication year range between 2019 and 2024, in English language and included in vitro articles on the mechanical properties of flexural strength, fatigue behavior, and microhardness of 3D-printed materials for temporary or definitive restorations. MeSH terms and free terms were used for the titles and abstracts of each article. Finally, the QUIN tool was used to assess the risk of bias. In the main search, 227 articles were found, of which 20 duplicates were excluded, leaving 207 articles; of these, titles and abstracts were read, and 181 that did not meet the eligibility criteria were eliminated; of the remaining 26 articles, 1 article was eliminated for not presenting quantitative results. Regarding publication bias, 6 of the 25 articles had a low risk of bias, 18 had a medium risk of bias, and 1 had a high risk of bias. It may be concluded that 3D-printed resins have lower flexural strength, fatigue behavior, and microhardness than other resin types used for the fabrication of temporary and permanent restorations. The type of 3D printer and polymerization time could be factors that significantly affect the flexural strength, fatigue behavior and microhardness of 3D-printed resins. Based on existing evidence, it should be considered that additive technology has promising future prospects for temporary and permanent dental restorations.

## 1. Introduction

The development of computer-aided design and manufacturing (CAD/CAM) systems has facilitated the fabrication of indirect restorations in a variety of materials, such as composite resin, which is used as an alternative in contemporary prosthetics [1,2,3]. In the last decade, dental restorations have mainly been fabricated using subtractive methods such as milling and grinding; however, these procedures have some disadvantages, such as material waste, dependence on the geometry of the milling instrument, and long processing time [1]. On the other hand, an additive manufacturing system has been developed using three-dimensional (3D) printing [2], which offers advantages such as reduced material consumption, lower heat and noise emissions, and the ability to manufacture elements with multiple, complex geometries simultaneously, reducing both manufacturing time and cost [3,4,5]. Despite the advantages of 3D printing in dentistry, there are some limitations to consider, such as polymerization shrinkage, the post-processing procedure, the calibration of the printer and the materials used, and the learning curve for professionals to use this technology [6].

Recently, new materials have been developed for the fabrication of indirect restorations by subtractive and additive methods, containing varying amounts of composite resin and some ceramic components in the same material, thereby integrating the beneficial characteristics of both composites [7]. In 2020, the first material for the fabrication of definitive tooth-colored indirect dental restorations (VarseoSmile Crown plus; BEGO, Bremen, Germany) was launched on the market. According to the manufacturer, it is suitable for the fabrication of crowns, inlays, onlays, and veneers. This material is a composite with ceramic particles consisting of a methacrylate matrix with ceramic filler (RMCs) [8]. Based on this material, other brands of printed resins with similar compositional characteristics have been marketed for definitive and temporary restorations. However, before these materials are recommended for routine clinical use, preclinical and clinical studies are needed to understand their long-term mechanical behavior [9].

For adequate clinical results, indirect restorations should follow biological, biomechanical, and esthetic principles. Mechanical properties for medium to long-term clinical success of the materials include flexural strength, microhardness, and fatigue resistance with the ability to withstand functional chewing forces without fracture or displacement [10]. It has to be considered that the mechanical properties of the material may deteriorate due to chewing processes and absorption of aqueous elements in the oral cavity after a long period of time. In addition, physical forces such as brushing may increase the surface roughness (Ra) with consequent discoloration of the restoration, wear of antagonist teeth, and retention of biofilm, which would increase the risk of gingivitis or secondary caries [11].

Currently, there are some in vitro studies in the literature on 3D-printed resins for definitive and temporary restorations that evaluate properties such as material strength, resilience, fracture toughness, microhardness, wear, surface roughness, among others, but there are just a few studies that evaluate these properties in the new resins [12,13,14]. Therefore, a systematic review of the current state of knowledge on the mechanical properties of 3D-printed resins for definitive and provisional dental restorations has been conducted. Therefore, the aim of this systematic review was to evaluate the mechanical properties of 3D-printed resins for indirect restorations according to published scientific evidence.

## 2. Methods

### 2.1. Protocol

This systematic review was conducted in accordance with the Preferred Reporting Items for Systematic Reviews and Meta-Analyses (PRISMA) guidelines [15]. It was registered in the OSF database (DOI: 10.17605/OSF.IO/NMH94) and can be found at https://doi.org/10.17605/OSF.IO/NMH94, accessed on 13 January 2025.

### 2.2. Literature Search

The search was conducted by two researchers, (DD) and (VB), in four digital databases: PubMed, EBSCO, Lilacs, and Science Direct. We searched for full-text articles with titles compatible with the research objectives, with a publication year limitation from 2019 to 2024 in the English language. The search strategy used MeSH terms in PubMed and free terms for the titles and abstracts of each article in the other digital databases, as well as a manual search of articles. Boolean operators, such as AND, OR, and NOT, were used. The researchers (DD and VB) conducted the search separately and, in case of disagreement on an article, the intervention of a third researcher (AC) and even a fourth (CAC) was requested until a consensus was reached. For the assessment of the risk of bias in in vitro studies, the QUIN tool was used, which consists of a questionnaire of 12 criteria specified below.

The keywords used to answer the PICO question are listed in Table 1.

### 2.3. Eligibility Criteria

In vitro articles that investigated the mechanical properties of flexural strength, fatigue behavior and microhardness of 3D-printed materials for temporary or final restorations were included. Review articles, case reports/series, those analyzing properties other than those mentioned in the aim of the present systematic review, and those not expressing the results numerically were excluded.

### 2.4. Criteria for Article Selection

**Figure 1:**
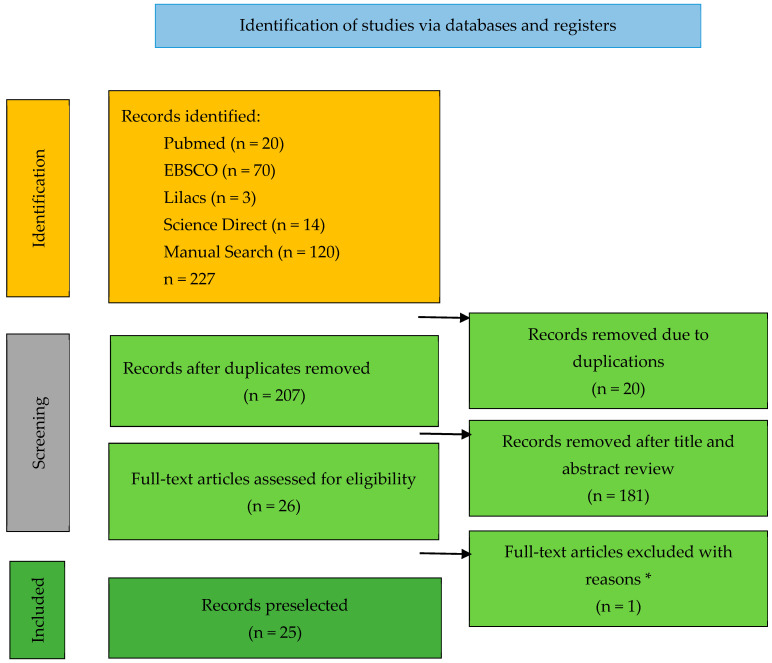
Flowchart of articles found. *: 1 Without quantitative results.

For the selection of the studies, two researchers (DD and VB) independently reviewed the studies found in the search of the digital databases, then proceeded to read the titles, excluding those that were not related to the topic of study, and then to read the abstracts to establish whether the studies met the inclusion criteria. Finally, each article selected by title and abstract was read in full text and the QUIN criteria (Figure 1) were applied to determine the risk of bias in order to assess the methodological quality of the articles with respect to their structure and execution. In the article selection process, any disagreement about the inclusion of any article was resolved by a third (AC) and even a fourth (CAC) researcher.

### 2.5. Selection, Management and Data Collection

Two reviewers (DD and VB) independently extracted data. Full-text articles selected for inclusion were managed using a standardized form in digital format (Office Excel 2016 software, Microsoft Corporation, Redmond, WA, USA). Information was collected on authors, year of publication, study design, sample size, materials evaluated, printer type, flexural strength methodology, microhardness, cyclic fatigue, results, conclusions, and risk of bias. A third and fourth reviewer (AC and CAC) were able to discern discrepancies when there was no agreement.

### 2.6. Assessment of Risk of Bias and Methodological Quality

For the assessment of the risk of bias in in vitro studies, the QUIN tool was used, which consists of a questionnaire with a list of 12 criteria: a. clearly stated objectives; b. sample size calculation; c. explanation of sampling technique; d. details of group comparison; e. explanation of methodology; f. operator details; g. randomization; h. method of outcome measurement; i. details of outcome assessment; j. blinding; k. statistical analysis; l. presentation of results. Each of these criteria was assigned a score of 2 if adequately specified, a score of 1 when insufficiently specified, and a score of 0 when not specified, and when those criteria were not applicable, they were excluded from the final calculation. Finally, the scores were summed to obtain a total score for each study assessed and assigned to high risk (<50%), medium risk (50% to 70%), and low risk (>70%) categories using the following formula:

Final score = (total score × 100)/(2 × number of applicable criteria)

The risk of bias of the studies included in the review was assessed independently, in duplicate, by two authors (DD and VB), and any disagreement in the assessment was resolved by consensus after the opinion of the third and fourth reviewers (AC and CAC).

### 2.7. Analysis and Synthesis of Data

There was a significant heterogeneity in the experimental design of the included articles, considering several factors, such as materials used, type of printer, layer thickness, orientation for printing, curing time, different statistical analyses, and other independent variables incorporated in the studies; so, it was not feasible to perform a meta-analysis of the quantitative data obtained in this review.

## 3. Results

### 3.1. Search and Selection

The selection process using the PRISMA flowchart is shown in Figure 1. The initial search yielded 227 articles, of which 20 studies were duplicated. Subsequently, 181 studies were excluded because their titles and abstracts did not meet the eligibility criteria. The full texts of the other 26 studies were reviewed, 1 of which did not mention specific numerical results for a proper comparative analysis and was therefore excluded from this study. All were in vitro studies (Table 2). Table 3, Table 4 and Table 5 show the flexural strength, fatigue behavior, and microhardness data classified according to the method used in each study: conventional, milled, and printed.

### 3.2. Assessment of Risk of Bias and Methodological Quality

Of the 25 in vitro studies included in this systematic review, 6 had a low risk of bias, 18 had a medium risk of bias, and 1 article had a high risk of bias (Table 6). The risks of bias found most frequently in the studies originated from the sample size calculation and the fact that the studies did not mention the number of operators who applied the different trials and whether they were blinded or not.

## 4. Discussion

The use of both temporary and permanent 3D-printed indirect dental restorations is increasing in the clinic due to the advantages of additive manufacturing [16]. However, the materials used for their manufacture must meet certain requirements, e.g., they must be biotolerable, biocompatible, and have suitable mechanical properties, such as high flexural strength, high microhardness, and good cyclic fatigue behavior [43]. Evaluation of these mechanical properties of 3D-printed restorations is essential to assess their structural integrity and suitability for clinical use [44]. Therefore, this systematic review aimed to evaluate the properties of restorations made with these technologies and materials based on additive systems.

Regarding the 3D-printing systems used to fabricate the restorations, of the studies evaluated in this systematic review, 15 of them used DLP printers, and 5 studies used stereolithography (SLA) technology printers [45]. On the one hand, DLP printers used high-power LED light to project in two dimensions (x/y axes), polymerizing the entire flat area of the construction at the same time and reducing working times [16]. In addition, the DLP printers achieved a high resolution, allowing for the production of dental restorations with high precision [44].

Another additive manufacturing method used FDM, where a liquefied filament is extruded from a nozzle, and the material is fused onto a scaffold. However, the resolution of DLP and SLA products has been found to be higher than that of FDM [46]. In one study, the flexural strength of 3D-printed resins manufactured from DLP, SLA, and FDM printers was compared using self-cure resin (CV) as a negative control group and milled resin (SM) as a positive control. The CV group had the lowest flexural strength (543 N), while the SLA group had the highest value (1323 N). No statistically significant difference was found in the flexural strength values between the DLP and SM groups (*p* = 0.481) and between the DLP and SM groups (*p* > 0.05), while the flexural strength of the SLA group showed statistically significant difference with the other groups (*p* < 0.001). The samples of the FDM group did not fracture, so it was impossible to determine the flexural strength value; however, it can be said that the material used for the samples of the FDM group had a higher elasticity [18].

An additional method of additive manufacturing found in this review was the selective laser sintering (SLS) of a selectively fused powder resin [47,48]. Meincke, D. et al. [23], in their study, compared the flexural strength, microhardness, and cyclic fatigue of 3D-printed provisional restorations fabricated using SLA and SLS techniques and compared them with conventional techniques (acrylic and bisacrylic resin). Regarding microhardness, a statistically significant difference was found in the evaluated groups, *p* < 0.001, where the acrylic resin presented the highest microhardness values (14.2 ± 2.6 Kgf/mm^2^), followed by bisacryl and SLS resin (10.7 ± 2.2; 10.3 ± 1.0, respectively) while the SLA-printed resin presented the lowest values (8.4 ± 0.2 Kgf/mm^2^). The flexural strength of the SLS resin (77.3 ± 3.1 MPa) was higher (*p* < 0.05), followed by bisacryl, acrylic, and finally SLA resin (75.0 ± 8.2, 69.2 ± 8.8, and 48.9 ± 1.2, respectively). Finally, SLA resin was the only material that fractured in the cyclic fatigue test.

There are several materials that can be used with 3D-printing technology, such as dental ceramics, composites, polymer resins like polyetheretherketone (PEEK) and PMMA, and metals like titanium, stainless steel, and Cr-Co alloys [44]. Currently, the vast majority of materials used in additive manufacturing are polymer-based materials, commonly known as 3DP, because of the method of manufacture [16,24]. Recently, 3D-printed composite resins have been launched on the market for the fabrication of individual definitive dental restorations marketed as resin-based hybrid composites (RBCs) [4,49].

In this systematic review, 18 studies compared the mechanical properties of 3D-printed resins with other types of materials. Atria, PJ. et al. [16] evaluated the biaxial flexural strength of four brands of 3D-printed resins (FL, CT, PB, and ND), where the PB resin showed the highest mechanical performance with statistically significant higher values (249.09 MPa) than the other resins tested. Aati, S. et al. [19] analyzed the flexural strength and microhardness of a 3D resin (C&B NextDent) reinforced with 1, 2, 3, 4, and 5% ZrO_2_ nanoparticles, where it was determined that the amount of nanoparticles concentration significantly affected the flexural strength (*p* < 0.01), improving the modified printed resin compared to the unmodified one. The maximum average flexural strength was around 111.59 MPa with 5% ZrO_2_, then decreased to a minimum value with unmodified resin of 98.32 MPa. Therefore, it can be determined that printed restorations still do not reach the values achieved in traditional materials, coinciding with a study carried out comparing the compressive strength of temporary restorations made in printed vs. milled resins [50]. In terms of microhardness, significant differences were observed between the unmodified resins and those reinforced with ZrO_2_ nanoparticles (*p* < 0.0001). No significant differences in microhardness were detected between the unmodified resin and the addition of 1%, 2%, and 3% or 3% and 4% ZrO_2_; the highest hardness was recorded for 5%. On the other hand, Karaoğlanoğlu, S. et al. [22] compared the microhardness of two 3D-printed resins (CT and PC) with two brands of milled resins (CS and GB), where the two brands of 3D-printed resins showed lower microhardness values (CT = 30.0 ± 1.3 MPa, PC = 37.4 ± 1.3 MPa) than the milled resins, and this result was statistically significant (*p* < 0.05). Similar results were found in other studies where the flexural strength of printed resins was lower than milled resins [31,33] but higher than conventional acrylic and bisacryl [18,23,35,41]. Regarding microhardness, in one study, printed resins had the lowest values [23], and conversely, in another study, microhardness values were higher than conventional methods [38]. However, the methodology used was diverse with Knoop and Vickers tests, so no absolute conclusions can be drawn.

Bora, P. et al. [24] evaluated the flexural strength and microhardness of four brands of 3D-printed resins (C&B, CC, OnX, and OnXT) with a light-curing composite resin, milled resin, and ceramic. For both properties, the printed resins obtained lower values than the rest of the materials. The highest value for flexural strength among the 3D-printed resins was obtained by the OnX group (131.0 ± 11.6 MPa) and the lowest by OnXT (78.0 ± 8.6 Mpa), while for microhardness, the highest value was obtained by the CC resin (42.5 ± 5.6 HV), and the lowest value was obtained by C&B (14.1 ± 0.6). With this same material (OnXT), lower results (1008 N) were obtained when compared to a PMMA milled material (2104 N), although the values were close to those that can be compatible for clinical practice for three-unit bridge restorations [51]. Similar results have been found in another study analyzed in the present investigation [1], where the biaxial flexural strength and cyclic fatigue of a 3D-printed resin was evaluated with a nanohybrid composite resin and a polymer infiltrated ceramic; the results showed a statistically significant difference between the groups for the two properties evaluated (*p* < 0.05), where the printed resin obtained the lowest values for biaxial flexural strength (83.5 ± 18.5 MPa) and cyclic fatigue (37.4 ± 23.8 MPa). This is in contrast to other studies, where the flexural strength of the printed resins was higher than a polymer-matrix infused ceramic but lower than the milled resin [29,30,36]. Abad et al. [26] compared the flexural strength of a 3D-printed resin with zirconium dioxide, porcelain fused to metal, and PMMA. PMMA had the highest mean strength values (2104.73 N), followed by PFM (1361.48 N), ZR O2 (1107.63 N), and finally 3DPP (1000.88 N); from these results, it can be observed that the lightest materials (PMMA and 3DPP) had the highest and lowest strength values, respectively. Finally, Abad, C. et al. [27] compared the flexural strength of two types of 3D-printed resins with milled PMMA. The highest flexural strength values were obtained for PMMA (1427 ± 36.9 N), followed by 3DPPa resin (1231.0 ± 380.1), and finally 3DPPb (1029.92 ± 166.4). A statistically significant difference (*p* < 0.05) was found between PMMA and the two types of resin.

It has been shown that the post-curing time can significantly affect the optical and mechanical properties of 3D-printed resins. Therefore, accurate adjustment of the exposure time is essential to obtain a balance between esthetics and mechanical strength in 3D-printed restorations [32]. Bayarsaikhana et al. [20], in their study, evaluated the flexural strength of a 3D-printed resin whose samples were post-cured at a time of 5, 15, and 30 min using four different 3D-printed post-curing chambers (LC, FC, CM, VE) and for 20, 40, and 60 s using a light curing (VA) lamp; the flexural strength was significantly higher in two 30 min LC and VE groups (140.15 and 134.87 MPa) than in the 5 min groups (119.31 and 114.71 MPa, respectively). The flexural strength did not differ significantly in all 30 min PCE groups and 20, 40, and 60 s VA groups (*p* > 0.05). As for microhardness, it was higher in all groups. When placed for 30 min post-curing, the 30 min CM and 30 min FC groups exhibited significantly higher hardness values of 16, 82 and 16, 4, respectively, with no significant differences between the 30 min LC and 60 s VA groups (*p* > 0.05). This finding contrasts with other research suggesting that post-curing using multi-spike LED curing units is not as effective as conventional devices, indicating that the post-curing method may influence the mechanical properties of resins [28]. Furthermore, it is reported that the flexural strength of 3D-printed resins can be markedly improved with as little as 5 min post-curing, and that, in many cases, times longer than 10 min do not generate significant changes in this property [32]. This suggests that, although the 30 min post-cure time used by Bayarsaikhana et al. showed positive results, it may be unnecessary to achieve optimal strength improvements, since other studies indicate that post-cure application improves the microhardness (KHN) of the materials evaluated without requiring extended periods [32].

Regarding the layer thickness, the results obtained in this study show that it significantly influences the mechanical properties of the resins used. In particular, it was observed that the group with a layer thickness of 100 μm presented the highest flexural strength compared to the thicknesses of 25 μm and 50 μm [34]. However, it is notable that all groups exceeded the minimum required flexural strength of 50 MPa for temporary crown materials, suggesting that these resins are suitable for clinical applications. In addition, the 50 μm group showed the highest average Vickers hardness, indicating that, although the 100 μm thickness offers advantages in strength, the 50 μm thickness provides superior microhardness. On the other hand, the fact that layer thickness and post-treatment conditions will not affect the degree of conversion of the printed material highlights the importance of other factors in optimizing mechanical properties [37]. Finally, the finding that varying layer thickness did not influence the flexural strength and Weibull characteristics of the interim material fabricated with the DLP printer suggests that, although layer thickness has an impact on certain properties, other aspects of the printing process and material also play a crucial role in the final performance of 3D-printed resins [40].

While we have described in this systematic review the application of 3D-printed resins in dentistry, there are other applications in terms of biomedicine. For example, it has been used to manufacture scleral, hand, and transtibial prostheses and to generate ankle foot, arm, and hand orthoses [52].

## 5. Conclusions

Based on the results of this systematic review, the following can be concluded:

- In general, 3D-printed resins showed lower flexural strength, fatigue behavior, and microhardness compared to other types of resin used for the fabrication of temporary and permanent restorations.
- The technology used, type of 3D printer, polymerization time, and post-processing processes are factors that significantly affect the flexural strength, fatigue behavior, and microhardness of 3D-printed resins.
- It has been shown that the main application of 3D-printed resins in prosthodontics is the manufacturing of dental crowns and bridges. Therefore, this technology would also have other applications in other fields of dentistry, such as implantology and orthodontics. It also can be applied in biomedicine within the manufacture of prothesis and orthoses.
- Finally, it should be considered based on the existing evidence that additive technology has promising future prospects for temporary and definitive dental restorations; so, further studies on this technology and materials should be conducted.

## Figures and Tables

**Table 1:** Digital databases and search strategy.

PUBMED	
P	(Crowns [MeSH Terms] OR Crowns [Title] OR Restorations [Title] OR Restorative [Title] OR Indirect Restorations [Title/Abstract] OR Fixed Dental Prostheses [Title/Abstract] OR Plural Fixed Prostheses [Title/Abstract] OR Single fixed prosthesis [Title/Abstract] OR Single Unit [Title/Abstract])
I	(3D-Printed [Title/Abstract] OR Three Dimensional Printed [Title/Abstract] OR 3-D Printing [MeSH Terms] OR 3-D Printing [Title/Abstract] OR Additive [Title/Abstract]) AND (Polymeric Material [Title/Abstract] OR Resin [Title] OR Composite Resins [MeSH Terms] OR Composite Resins [Title/Abstract] OR Restoration Material [Title])
C	1 2 and 3
O	(Flexural Strength [MeSH Terms] OR Flexural Strength [Title/Abstract] OR Fatigue Behavior [Title/Abstract] OR Microhardness [Title/Abstract] OR Fracture Strength [Title/Abstract] OR Fracture Resistance [Title/Abstract] OR Mechanical Properties [Title/Abstract] OR Flexure [Title])
EBSCO	
P	(TI Crowns) OR (TI Indirect Restorations) OR (TI Fixed Dental Prostheses) OR (TI Plural Fixed Prostheses) OR (TI Restorations) OR (TI Restorative)
I	(TI 3D Printed) OR (TI Three Dimensional Printed) OR (TI 3D Printing) OR (TI Aditive) OR (TI Polymeric Material) OR (TI Resin) OR (TI Composite Resins)
C	1 2 and 3
O	(TI Flexural Strength) OR (TI Fatigue Behavior) OR (TI Microhardness) OR (TI Fracture Strength) OR (TI Fracture Resistance) OR (TI Mechanical Properties)
LILLACS	
P	(ti:(“Crowns”)) OR (ti:(“Plural Fixed Prostheses”)) OR (ti:(“Indirect Restorations”)) OR (ti:(“Fixed Dental Prostheses”)) AND (“Restorations”)
I	(TI “3D Printed”) OR (“TI Three Dimensional Printed”) OR (TI “3D Printing”) OR (TI “Aditive”) OR (TI “Polymeric Material”) OR (TI “Resin”) OR (TI “Composite Resins”)
C	1 2 and 3
O	(TI:(“Flexural Strength”)) OR (TI:(“Fatigue Behavior”)) OR (TI:(“Microhardness”)) OR (TI:(“Fracture Strength”)) OR (TI:(“Fracture Resistance”)) AND (TI:(“Mechanical Properties”))
SCIENCE DIRECT	
P	Title, abstract, keywords: “Crowns” OR “Indirect Restorations”
I	Title, abstract, keywords: “3D Printed” OR “Three Dimensional Printed” OR “Resin” OR “Composite Resins”
C	1 2 and 3
O	Title, abstract, keywords: “Flexural Strength” OR “ Microhardness” OR “Fatigue Behavior”

**Table 2:** Demographic and 3D printer characteristics for included studies.

Demographics Characteristics						Printer Parameters				
ID Study	Year	SS	ST&ISO	Property	PT&M	RT&B	LT (μm)	CG (°)	PCT	Control
[16]	2022	30	1.2 mm thick and 14 mm diameter disc ISO 6872:2024 [17]	Biaxial FS	SLA Formlabs, Somerville, MA, USA DLP NextDent 5100; 3D Systems, Soesterberg, NL	1. Crowntec (CT) 2. Permanent bridge resin (PB) 3. Formlabs (FL) 4. NextDent (ND)	50	NA	FL: 20 min CT: 6 min PB: 6 min ND: 30 min	NA
[18]	2020	15	Three-unit plural fixed prosthesis	FS	DLP NextDent 5100; Soesterberg, NL SLA Formlabs, Somerville, MA, USA, FDM FlashForge	1. DLP: PMMA (D-150 NextDent) 2. SLA: Form2 Formlabs 3. FDM: Polylactic acid (Creator pro, FlashForte)	SLA and DLP: 100 FDM: 200	30°	DLP: 120 min SLA: 60 min, FDM did not undergo post-curing	Self-curing (CV) as negative control: PMMA Jet Tooth (ShadeTM Powder, Lang Dental Co., Wheeling, IL, USA). Subtractive method (SM) as positive control: PMMA (ViPi, VIPI Co., Sao Paulo, Brazil)
[19]	2021	15	Discs of 15 mm diameter and 1 mm thickness	FS and MH	DLP Kulzer, Australia	C&B NextDent 3D resin reinforced with ZrO_2_ nanoparticles at 1, 2, 3, 4 and 5%	50	NA	20 min	C&B NextDent 3D resin without reinforcement
[20]	2022	FS: 25 MH: 5	FS: Bar with dimensions of 2 mm × 2 mm × 25 mm ISO: 10477 [21] MH: Discs with a diameter of 10 mm and a thickness of 3 mm	FS and MH	DLP NextDent 5100; Soesterberg, NL	C&B NextDent 3D resin.	50	NA	5, 15, and 30 min with LC 3D Print Box (LC), Form Cure (FC), Cure M (CM) and Veltz 3D (VE) 20 s, 40 s, and 60 s with Valo (VA)	Specimens of the group that were not subjected to post-curing
[22]	2023	96	12 × 8 × 2 mm blocks	MH	SLA Asiga MAX UV, Australia	Permanent 3D resins: Crowntec (Saremco Dental AG) (CT) Permanent Crown (Formlabs) (CP)	50	NA	6 and 20 min	CAD/CAM blocks based on Cerasmart (CS) resin and Grandio Blocs (GB)
[23]	2022	MH: 5 FS: 10	FS: 4 × 2 × 10 mm bars MH: 10 × 2 mm diameter discs CF: individual crowns	FS, MH and CF	SLA and SLS	SLA 3D-printed resin SLS 3D-printed resin	NA	NA	SLA: 30 min SLS: 0 min	Acrylic resin (RA) and bisacrylic resin (BIS)
[24]	2024	14	FS: 2 mm × 2 mm × 25 mm bars MH: 4 × 4 × 6 mm blocks ISO: 4049 [25]	FS and MH	DLP Pro 55, SprintRay, LA, CA	C&B 3D-printed resin MFH (C&B) Ceramic Crown (CC) SprintRay OnX (OnX) SprintRay OnX Tough (OnXT)	100	NA	NA	Light-curing composite resin Filtek Supreme Ultra (FS), Lava Ultimate milled composite resin (LU) and IPS e.max CAD milled ceramic (e.max)
[26]	2023	8	4-unit plural fixed prosthesis	FS	DLP Pro 95, SprintRay, Los Angeles, CA, USA	3DPP (Sprintray)	NA	NA	NA	Zirconium dioxide (ZR O2) (KATANA, Zirconia STML), Porcelain fused to metal (PFM) and PMMA (Telio CAD, Ivoclar Vivadent)
[1]	2024	FS: 30 CF: 20	14 mm Disc with a diameter of 15 mm and a height of 1.5 mm ISO: 6872	Biaxial FS and CF	DLP Varseo XS, Bego	VarseoSmile Crown Plus (3D)	50	NA	NA	Nanohybrid composite resin (NHC group) (Grandio, VOCO) and polymer-infiltrated ceramic (PICN group) (Enamic, Vita Zahnfabrik)
[27]	2023	20	Unitary fixed prothesis	FS	DLP Pro 95, SprintRay, Los Angeles, CA, USA	3DPPa (SprintRay) 3DPPb (SprintRay)	50	NA	9 min	Milled PMMA (Ivoclar Vivadent)
[28]	2024	MH: 792 FS: 180	MH: Discs FS: Bar-shaped specimens	FS and MH	NA	NextDent C&B MFH	NA	NA	Fast and Standard modes with VALO (V1 and V2) and BluePhase (B1 and B2) cured units and Conventional post curing (PC group)	NA
[29]	2023	6	NA	FS MH	NA	Varseo Smile Crown plus-(VSC) Saremco Print Crowntec (SPC) Formlabs 3B Permanent crown (FLP)	NA	NA	NA	Vita Enamic -VE, Cerasmart -CE, Lava Ultimate-LU
[30]	2023	10	NA	FS	NA	3D-printed VarseoSmile Crown Plus (VSC)	NA	NA	NA	Milled Vita Enamic (VE), milled Cerasmart 270 (CS)
[31]	2021	Total: 368	MD: 10 × 10 × 4 mm bars RF: 2 × 3 × 15 mm bars	FS and MH	fused filament fabrication (FFF)	Polyphenylenesulfone (PPSU): PPSU1-3D = PPSU Radel PPSU2-3D = Ultrason P 3010 NAT	NA	NA	NA	Polyetheretherketone semi-crystalline (PEEK- CG) milled and PPSU obtained by extrusion (PPSU1- EX)
[32]	2022	RF: 12 MD: 10	RF: 2 × 2 × 2 mm bars ISO: 4049 MD: 5 × 5 × 5 mm blocks	FS and MH	DLP Photon, Anycubic Technology Co., Shenzhen, China	Cosmos Temp3D (COS), SmartPrint BioTemp (SM) Resilab3D Temp (RES) and Prizma3D BioProv (PRI)	50	0°	5 min 10 min 15 min 20 min	NA
[33]	2023	30	8 × 2 × 2 mm bars ISO: 6872:2024	FS	DLP Miicraft Ultra 125	Temp 3D-printed resin (PRINT)	65	90°	7 min	Acrylic resin Dencor (AR), Filtek Z350XT Composite Resin (CR), VIPI Milled PMMA Resin (CAD) and Bisacril Protemp 4 (BIS)
[34]	2023	16	2 × 2 × 25 mm bars ISO: 10477	MH	DLP NextDent 5100; Soesterberg, NL Asiga MAX Nova 3D Master, Australia	1. NextDent 3D-printed resin 2. Asiga DentaTooth 3. JamgHe	50	1. 0° 2. 90°	NA	NA
[35]	2020	MD: 8 RF: 10	diameter and 2 mm height discs	FS and MH	SLA NextDent 5100; Soesterberg, NL	Crown & Bridge 3D-printed resin, MFH (Next Dent) (PR)	NA	NA	NA	Bisaccharide resin ProTemp Plus (3M ESPE) (BA) and Filtek Z350XT Conventional Composite Resin (3M ESPE) (Z350)
[36]	2021	10	15 × 4 × 1.5 mm bars	FS and MH	Sonic Mini 4K Phrozen, Hsinchu City, Taiwan	VarseoSmile Crown plus ^®^ 3D-printed resin (Bego)(VSC)	50	1. 90° 2. 45°	2 times of 45 min	Grandio pads (VOCO) (GR), Brilliant Crios^®^ (Coltene/Whaledent AG Altstatten)—(CR), Enamic^®^ (Vita Zahnfabrik)—(EN)
[37]	2022	210	25 × 2 × 2 mm bars	FS and MH	DLP Everes zero, SISMA, Italy	A2 EVERES TEMPORARY printed resin, SISMA, Italy	25, 50, 100	90°	LC: 5 and 15 min HC: 5 and 15 min	NA
[38]	2021	20	Discs of 10 mm diameter and 2 mm thickness	MH	1. Vat-polymerization 3D printer Rapidshape D30; Rapidshape, Heimsheim, Germany. 2. Vat-polymerization printer Envisiontec VIDA; Dearborn, MI, USA	Printed Resin: 1. AM-1 (FreePrint temp; Detax) 2. AM-2 (E-Dent 400 C&B MFH; Envisiontec) 3. AM-3 (NextDent C&B MFH; 3D Systems), 4. AM-4 (Med620 VEROGlaze; Stratasys).	50	90°	1: 6 min 2: 15 min 3: 30 min 4: NA	Conventional Materials: CNV-1 (Protemp 4; 3M ESPE) CNV-2 (Anaxdent new outline dentin; Anaxdent),
[39]	2022	20	Rectangular samples (2 × 2 × 25 mm). ISO: 10477	FS and MH	DLP NextDent 5100; Soesterberg, NL	Photopolymer (Crown & Bridge NextDent^®^; 3D Systems, Soesterberg, Countries Low)	50	0°	30 min	Self-healing provisional material (Bosworth Trim Plus; Bosworth, Skokie, IL, USA) and prefabricated resin blocks (Ceramill temp; Amann Girrbach AG, Koblach, Austria)
[40]	2023	20	25 × 2 × 2 mm bars	FS	Asiga MAX UV, Australia	Temporary resin (Nexdent C&B MFH)	10, 25, 75, 100, 125 and 150	NA	30 min	Control group 50 um
[41]	2023	15	Discs 10 × 2 mm	FS	3D Printer EPAX, Morrisville, NC, USA	MFH (NextDent C&B)	NA	NA	30 min	conventional (Protemp 4, Tuff-Temp, Tempron), CAD-CAM milling (VITA CAD-Temp, breCAM.multiCOM)
[42]	2023	196 samples	25 × 2 × 2 mm ISO: 10477	FS	1. DLP Pro 95, SprintRay, Los Angeles, CA, USA 2. SLA Form 3, Formlabs, Somerville, MA, USA	3D printing resins 1. UDMAC 2. BEMAC	NA	NA	UDMA: 20 min BEMA: 30 min	NA

SS: Sample size per group. ST&ISO: Sample type and ISO standard applied. PT&M: Printer Type/Model. RT&B: Resin Type/Brand. LT: Layer thickness. CG: Construction guidance. PCT: Post-curing time. DLP: Digital light projection. FS: Flexural Strength. MH: Microhardness. CF: Cyclic fatigue.

**Table 3:** Evaluation of flexural strength.

**Conventional Method**					
**Material**	**Brand**	**FS (MPa)**	**DS**	**FS (N)**	**DS**
Bisacryl resin	Protemp [35]	27.9	±6.10		
UDM resin	Tuff Temp [41]	48.95	±87.64		
PMMA	Tempron [41]	61.43	±7.29		
Acrylic resin	Dencor, Brazil [23]	69.2	±8.8		
Bisacryl resin	Yprov Bisacryl [23]	75.0	±8.2		
PMMA	Bosworth [39]	76.0	±12		
Composite	Z350 Filtek (3M) [33]	84.0	±18.54		
Acrylic resin	Dencor, Brazil [33]	89.6	±9.38		
Composite	Z350 Filtek (3M) [35]	105.1	±9.80		
Bisacryl resin	Protemp 4 [41]	113.06	±14.45		
Bisacryl resin	Protemp [33]	118.23	±16.26		
Composite	Z350 Filtek 3M [24]	156.9	±14.8		
PMMA	Lang dental [18]			543 N	
Polylactic acid	Pla ColorFabb [18]			1323 N	
**Milled Method**					
**Material**	**Brand**	**FS (MPa)**	**DS**	**FS (N)**	**DS**
PMMA	VITA CAD-Temp [41]	62.48	±5.90		
PMMA	breCAM.multiCOM [41]	77.88	±10.25		
PMMA	Amann [39]	94	±19		
PMMA	VIPI [33]	94.63	±9.89		
Resin	Cerasmart 270 [22]	109.5	±1.9		
Resin	Enamic VITA [36]	118.96			
Resin	Enamic VITA [1]	140.3	±12.9		
Resin	Brilliant blocs [36]	170.29			
Resin	Lava ultimate (3M) [24]	183.6	±17.5		
Resin	Grandio blocs [36]	186.02			
Resin	Grandio blocs [22]	203.9	±3.6		
Resin	Grandio blocs [1]	237.3	±31.6		
Ceramic	E.max Ips [24]	299.3	±26.0		
Resin	Vita Enamic [30]			727.8 N	
Resin	Cerasmart 270 [30]			1213.8 N	
PMMA	Telio CAD [26]			2104.73 N	
**3D-Printed Method**					
**Material**	**Brand**	**FS (MPa)**	**DS**	**FS (N)**	**DS**
Resin	Cosmos Temp 3D [32]	19.5	±2.7		
Resin	Smart Print Bio Temp [32]	21.9	±2.1		
Resin	Prizma 3D [32]	33.7	±4.3		
Resin	Resilab 3D Temp [32]	34.2	±3.7		
Resin	Cosmos Temp 3D [33]	49.7	±7.55		
PMMA	C&B NextDent [35]	67.15	±11.70		
Resin	OnXT (SprintRay) [24]	78	±8.6		
Resin	Eves temporary [37]	80.8			
Resin	Varseo Smile Crown Plus [1]	83.5	±18.5		
PMMA	C&B NextDent [19]	94.14			
PMMA	C&B NextDent [24]	97.1	±4.6		
PMMA	C&B NextDent [41]	100.87	±11.14		
Resin	Veltz 3D [20]	110			
PMMA	C&B NextDent [39]	114	±8		
Resin	Ceramic Crown (Sprint Ray) [24]	117.4	±11.6		
Resin	Varseo Smile Crown Plus [36]	119.85			
Resin	PrintBox [20]	120			
Resin	Permanent Crown, Formlabs [42]	128	±22.4		
Resin	Form Cure [20]	130			
Resin	OnX (Sprint Ray) [24]	131	±11.6		
Resin	Cure M [20]	139			
Resin	Tera Harz TC-80DP, Graphy [42]	143.6	±13.1		
PMMA	C&B NextDent [16]	153.51			
Resin	Crowntec [16]	187.73			
Resin	Permanent bridge [16]	208.03			
Resin	Formlabs [16]	249.09			
PMMA	C&B NextDent [40]	296.6	±11.97		
Resin	PA2201; Stratasys Direct Manufacturing	452.4	±35.8		
Resin	Formlabs [23]	513.3	±29.7		
Resin	Sprint Ray [26]			1000.88	
Resin	Nano ceramic hybrid (Sprint Ray) [27]			1029.92	±166.4
Resin	Varseo Smile Crown Plus [30]			1181.5	
PMMA	C&B NextDent [18]			1189	
Resin	Hybrid material (SprintRay) [27]			1231	±380.1
PMMA	Formlabs [18]			1323	
PMMA	Ivoclar [27]			1427	±36.9
PSSU	Radel R-5000 NT			83 *	
PSSU	PPSU Radel			78.8 *	
PSSU	Ultrason P 3010 NAT			158.1 *	
PEEK	PEEK Juvora			139.1 *	

FS: Flexural strength. MPa: Megapascal. N: Newtons. DS: Standard deviation. PMMA: Polymethyl methacrylate. PEEK: semi-crystalline poly-etheretherketone. PPSU: amorphous polyphenylene sulfone. * Values represented in N/mm^2^.

**Table 4:** Evaluation of microhardness.

**Conventional Method**			
**Material**	**Brand**	**MH** **(Kgf/mm^2^)**	**DS**
Acrylic resin	Dencor [23]	14.2	±2.6
PMMA	Bosworth [39]	19.1	
Bisacryl	Protemp [35]	22.1	±3.10
Composite	Z350 Filtek 3M [35]	61.7	±5.70
Composite	Z350 Filtek 3M [24]	91.5	±10.4
Bisacryl	Protemp [38]	4.92 *	±0.36
Acrylic resin	Next outline Anaxdent	13.35 *	±5.84
**Milled Method**			
**Material**	**Brand**	**MH** **(Kgf/mm^2^)**	**DS**
PMMA	Amann [39]	24.3	
Resin	Brilliant crios [36]	75.4	
Resin	Lava ultimate 3M [24]	114.8	±28.1
Resin	Grandio blocs [36]	140.43	
Resin	Enamic [36]	273.42	
Ceramic	E.max IPS [24]	574	±29.0
**3D-Printed Method**			
**Material**	**Brand**	**MH** **(Kgf/mm^2^)**	**DS**
Resin	Cosmos Temp 3D [32]	4.58	±0.59
Resin	Resilab 3D TEMP [32]	7.46	±0.60
Resin	Smart Print Bio [32]	8.37	±0.93
Resin	Formlabs [23]	8.4	±0.2
Resin	JamgHe temporary resin, Nova 3D Master [34]	10	
Resin	Prizma 3D [32]	10.22	±0.68
Resin	PA2201; Stratasys Direct Manufacturing [23]	10.3	±1.0
PMMA	C&B Next Dent [24]	14.1	±0.6
Resin	Everes temporary [37]	14.33	
PMMA	C&B Next Dent [20]	16	
PMMA	C&B Next Dent [19]	17.39	
Resin	OnXT [24]	17.6	±0.8
Resin	DentaTooth, Asiga [34]	23.4	
PMMA	C&B Next Dent [34]	24.5	
PMMA	C&B Next Dent [39]	25.2	
Resin	Varseo smile crown [36]	25.8	
Resin	OnX [24]	29.3	±2.1
Resin	Crowntec [22]	30	±1.3
PMMA	C&B Next Dent [35]	35	±2.50
Resin	Varseo smile crown, Saremco Print Crowntec, Formalbs Permanent Crown [29]	35.11	±4.46
Resin	Permanent crown [22]	37.4	±1.3
Resin	Ceramic Crown [24]	42.5	±5.6
Resin	Vita Enamic, Cerasmart, Lava Ultimate [29]	253.5	±21.5
PMMA	C&B Next Dent [38]	9.91 *	±3.71
Resin	Free Print Temp [38]	12.55 *	±2.93
Resin	E Dent 400 C&B MFH [38]	13.03 *	±3.29
Resin	VeroGlaze MED620 [38]	13.45 *	±2.93
PSSU	Radel R-5000 NT	111 **	
PSSU	PPSU Radel	113 **	
PSSU	Ultrason P 3010 NAT	121 **	
PEEK	PEEK Juvora	207 **	

MH: Microhardness. DS: Standard deviation. PMMA: Polymethyl methacrylate. PEEK: semi-crystalline poly-etheretherketone. PPSU: amorphous polyphenylene sulfone. * Values represented in KHN. ** Values represented in N/mm^2^.

**Table 5:** Evaluation of cyclic fatigue.

**Milled Method**			
**Material**	**Brand**	**CF (MPa)**	**DS**
Resin	Enamic [1]	73.5	±9.9
Resin	Grandio blocs [1]	141.3	±3.8
**3D-Printed Method**			
**Material**	**Brand**	**CF (MPa)**	**DS**
Resin	Varseo smile crown [1]	37.4	±23.8

CF: Cyclic fatigue. MPa: megapascal. DS: Standard deviation.

**Table 6:** Risk of bias results.

Number	Author	Year	Study	Criteria QUINN and Points												Total (%)	Bias Risk
				1	2	3	4	5	6	7	8	9	10	11	12		
1	Atria PJ, et al. [16]	2021		2	0	NA	1	2	NA	NA	2	NA	0	2	2	11 (68.75%)	Medium
2	Park SM. et al. [18]	2020	in vitro	2	0	NA	2	2	NA	NA	1	NA	0	2	2	11 (68.75%)	Medium
3	Aati S, et al. [19]	2021	in vitro	2	0	NA	2	2	NA	NA	1	NA	0	1	2	10 (62.5%)	Medium
4	Bayarsaikhan E. et al. [20]	2022	in vitro	2	0	NA	2	2	NA	NA	2	NA	0	2	2	12 (75%)	Low
5	Karaoglandoglu S, et al. [22]	2023	in vitro	2	2	NA	0	2	NA	NA	0	NA	0	1	2	9 (56.25%)	Medium
6	Simoneti DM. et al. [23]	2022	in vitro	2	0	NA	1	1	NA	NA	0	NA	0	1	2	7 (43.75%)	High
7	Bora PV. et al. [24]	2024	in vitro	2	2	NA	2	2	NA	NA	2	NA	0	2	2	14 (87.5%)	Low
8	Abad Coronel C, et al. [26]	2023	in vitro	2	0	NA	0	2	NA	NA	2	NA	0	2	2	10 (62.5%)	Medium
9	Prause E. et al. [1]	2024	in vitro	2	0	NA	2	2	NA	NA	1	NA	0	2	2	11 (68.75%)	Medium
10	Abad Coronel C, et al. [27]	2023	in vitro	2	0	NA	1	2	NA	NA	2	NA	0	2	2	11 (68.75%)	Medium
11	Chung SH, et al. [28]	2024	in vitro	2	0	NA	2	2	NA	NA	2	NA	0	1	2	11 (68.75%)	Medium
12	Sahin Z, et al. [29]	2023	in vitro	2	0	NA	2	2	NA	NA	2	NA	0	1	2	11 (68.75%)	Medium
13	Abdulkareem MA, et al. [30]	2023	in vitro	2	0	NA	2	2	NA	NA	2	NA	0	1	2	11 (68.75%)	Medium
14	Schönhoff LM, et al. [31]	2021	in vitro	2	0	NA	1	1	NA	NA	1	NA	0	2	2	9 (56.25%)	Medium
15	Soto-Montero J, et al. [32]	2022	in vitro	2	0	NA	0	2	NA	NA	1	NA	0	2	2	9 (56.25%)	Medium
16	Ribeiro AKC, et al. [33]	2022	in vitro	2	0	NA	1	2	NA	NA	1	NA	0	2	2	10 (62.5%)	Medium
17	Alageel O, et al. [34]	2023	in vitro	2	2	NA	1	2	NA	NA	1	NA	0	2	2	12 (75%)	Low
18	Scotti CK, et al. [35]	2020	in vitro	2	0	NA	2	2	NA	NA	2	NA	0	2	2	12 (75%)	Low
19	Grzebieluch W, et al. [36]	2021	in vitro	2	0	NA	1	2	NA	NA	2	NA	0	2	2	11 (68.75%)	Medium
20	Alshamrani AA, et al. [37]	2022	in vitro	2	0	NA	2	2	NA	NA	2	NA	0	1	2	11 (68.75%)	Medium
21	Revilla-León M, et al. [38]	2021	in vitro	2	1	NA	1	1	NA	NA	2	NA	0	1	2	10 (62.5%)	Medium
22	Alageel O, et al. [39]	2022	in vitro	2	1	NA	1	2	NA	NA	2	NA	0	2	2	12 (75%)	Low
23	Scherer M, et al. [40]	2023	in vitro	2	1	NA	1	2	NA	NA	2	NA	0	1	1	10 (62.5%)	Medium
24	Sadek HMA, et al. [41]	2023	in vitro	2	1	NA	1	2	NA	NA	2	NA	0	2	2	12 (75%)	Low
25	Kang YJ, et al. [42]	2023	in vitro	2	0	NA	1	2	NA	NA	2	NA	0	2	2	11 (68.75%)	Medium
